# MLNet: a multi-level multimodal named entity recognition architecture

**DOI:** 10.3389/fnbot.2023.1181143

**Published:** 2023-06-20

**Authors:** Hanming Zhai, Xiaojun Lv, Zhiwen Hou, Xin Tong, Fanliang Bu

**Affiliations:** ^1^School of Information Network Security, People's Public Security University of China, Beijing, China; ^2^Institute of Computing Technology, China Academy of Railway Sciences Corporation Limited, Beijing, China

**Keywords:** multimodal named entity recognition, short text, multi-head attention, pre-training, cross task

## Abstract

In the field of human–computer interaction, accurate identification of talking objects can help robots to accomplish subsequent tasks such as decision-making or recommendation; therefore, object determination is of great interest as a pre-requisite task. Whether it is named entity recognition (NER) in natural language processing (NLP) work or object detection (OD) task in the computer vision (CV) field, the essence is to achieve object recognition. Currently, multimodal approaches are widely used in basic image recognition and natural language processing tasks. This multimodal architecture can perform entity recognition tasks more accurately, but when faced with short texts and images containing more noise, we find that there is still room for optimization in the image-text-based multimodal named entity recognition (MNER) architecture. In this study, we propose a new multi-level multimodal named entity recognition architecture, which is a network capable of extracting useful visual information for boosting semantic understanding and subsequently improving entity identification efficacy. Specifically, we first performed image and text encoding separately and then built a symmetric neural network architecture based on Transformer for multimodal feature fusion. We utilized a gating mechanism to filter visual information that is significantly related to the textual content, in order to enhance text understanding and achieve semantic disambiguation. Furthermore, we incorporated character-level vector encoding to reduce text noise. Finally, we employed Conditional Random Fields for label classification task. Experiments on the Twitter dataset show that our model works to increase the accuracy of the MNER task.

## 1. Introduction

In the field of human–computer interaction, a large number of novel techniques have been proposed to improve efficiency, reduce operational difficulty, and increase recognition accuracy. Currently, natural language-based human–robot interaction has been widely used (Ahn et al., 2018; Park et al., 2019; Walker et al., 2019). At the same time, the field of image perception is also developing rapidly. The combination of visual information and natural language can further improve the service response capability of robots. Especially when robots need to perform tasks related to entity features, it is meaningful to introduce multimodal named entity recognition techniques. The human-generated text that the robot needs to process is often spoken and noisy, which is somewhat similar to the characteristics of free posting on social media. Therefore, we choose the corpus and images from social media postings for the named entity recognition task, thus helping to improve multimodal human–robot interaction.

With the widespread use of social media platforms, the number of individual user postings has grown rapidly. Such interesting and diverse informal expressions provide users with rich information while providing a large amount of raw corpus data for natural language processing (NLP). Named Entity Recognition (NER), as a precursor to many information extraction tasks, aims to discover multiple categories of named entities, such as Person (PER), Location (LOC), and Organization (ORG), from raw text data. Given its importance, NER has attracted significant attention in the research community (Lample et al., 2016; Jiang et al., 2021; Radmard et al., 2021; Tian et al., 2021).

**Figure 1:**
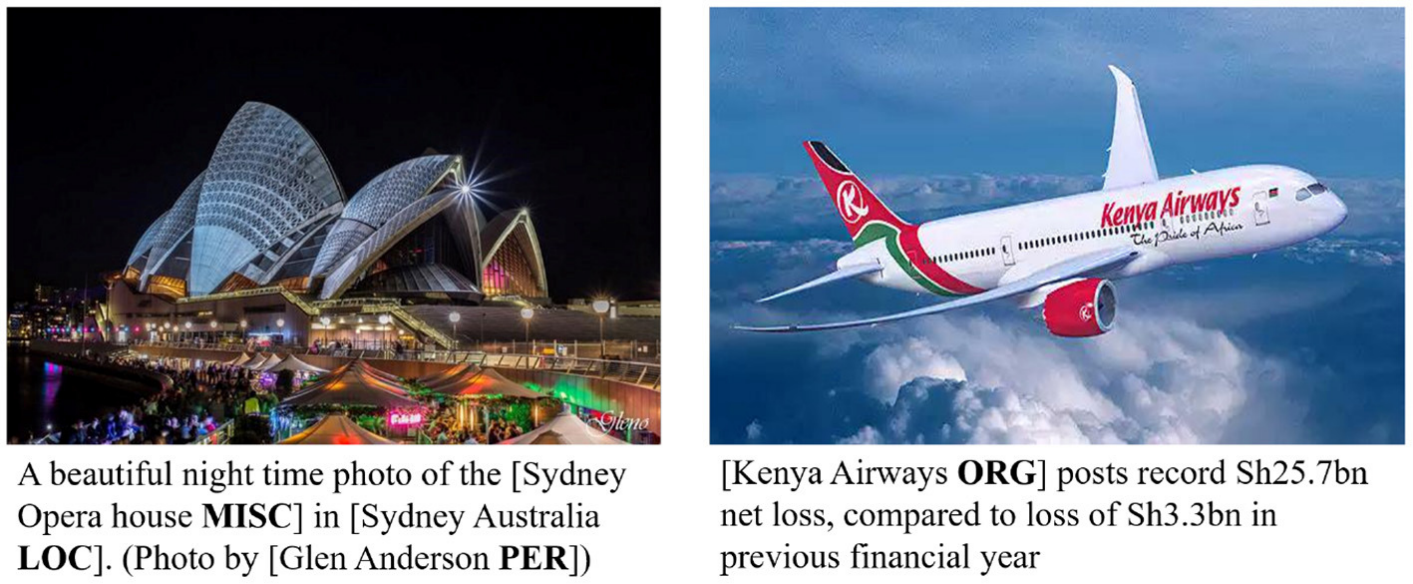
Two examples for multimodal named entity recognition.

Although a large number of excellent methods have emerged that enable increasing accuracy in named entity recognition efforts, most of them are based on news report texts or domain-length texts (Li et al., 2021; Wang et al., 2021, 2022). When solving named entity recognition tasks for social media texts (e.g., tweets), their shorter length and large amount of noise are fully considered, and thus, their performance is often much lower than that in news report texts. In general, common text noise in tweets includes misspellings, web abbreviations, and some informal newly invented words. In recent years, the posting format of social media platforms has been innovated, and the “text-image” format has gradually become mainstream, and some studies have proposed using visual features to improve the performance of NER (Arshad et al., 2019; Asgari-Chenaghlu et al., 2020; Chen et al., 2022). In this study, we will focus on multimodal named entity recognition (MNER) for social media postings, with the goal of extracting the corresponding entities from “text-image” pairs. This is shown in Figure 1.

A large number of excellent algorithms for multimodal named entity recognition already exist (Moon et al., 2018; Chen D. et al., 2021), but there are still some problems as follows: (1) The expressions in social media posts are often informal, colloquial, and even have certain spelling errors, which can affect the accuracy of text recognition. (2) Since short texts in social media contain less contextual information, there is some difficulty in determining entity types, which requires the use of image information to achieve semantic disambiguation. (3) For image information screening and fusion problems, there may be a large amount of irrelevant information in the whole picture, and there is some interference in entity extraction. Even the accompanying images in some posts may be irrelevant to the text, resulting in lower accuracy when directly fusing image features with text.

To solve these problems, we will pre-train the image and text data separately to extract the focused object features in the image and the word embedding and character embedding in the text as the input. We propose a multi-modal BERT model that uses a filtering gate to preserve visually relevant image features and trains an adaptive attention network to fuse image and textual features. Specifically, we design a symmetrical image-text fusion module that fully integrates multi-modal data by exchanging filtered feature information bi-directionally. To address the limited expressiveness and noise issues of short textual information, we further enhance the understanding of textual features using a bidirectional long short-term memory network. Finally, we use a conditional random field for label classification and complete the entity recognition task. We conduct experiments on the Twitter dataset to validate the effectiveness of our proposed model.

The main contributions of this study are as follows.

1. Based on a more efficient multimodal encoding scheme to reduce the noise in social media posts and improve the accuracy of named entity recognition tasks in informal corpus;
2. Proposes a new symmetric unified architecture for named entity recognition based on image-text features for multi-channel and multi-level input computation;
3. Achieves better performance on the Twitter2015 and Twitter2017 datasets.

## 2. Related work

### 2.1. Named entity recognition

NER has been attracting a lot of attention from the research community as a precursor to many natural language processing (NLP) tasks. While traditional NER tasks often rely on specific knowledge and manual annotation combined with statistical learning methods, with the continuous development of neural networks, most NERs are now opting for deep learning approaches. Various supervised learning-based approaches have been proposed, focusing primarily on designing the structure of neural networks so that more valuable features are fed into the classifier. The first use of neural networks applied to the study of named entities was by Hammerton (2003). They used a one-way long short-term memory network (LSTM), which has good sequence modeling capabilities, and LSTM-CRF became the underlying architecture for entity recognition. Later, based on this model, Lample et al. (2016) proposed a neural network model combining Bidirectional Long Short-Term Memory (BiLSTM) and Conditional Random Fields (CRF), a bidirectional structure capable of acquiring contextual sequence information. Pinheiro and Collobert (2014) first applied the combination of CNN and CRF structures in named entity recognition research with good results on CoNLL-2003. In the field of chemistry, Luo et al. (2018) used the BiLSTM-CRF model based on the attention mechanism to further improve the performance of entity recognition.With the introduction of the BERT model, more research has focused on the improvement and optimization of the pre-trained model, and the effectiveness of the NER task has been further improved (Devlin et al., 2018; Jawahar et al., 2019; Souza et al., 2019). The combination of BERT as an encoder with LSTM-CRF by Liu et al. (2019) and Luo et al. (2020) and the combination of BERT and a threshold control unit (GRU) by Liu et al. resulted in better results for the NER task on the CoNLL-2003 dataset.

However, these methods tend to be more suitable for NER tasks in formal texts, and most of them do not achieve satisfactory results when facing social media texts. To address this problem, many studies have added some resources other than text to the input of NER, which can achieve better performance on social media texts. Moreover, as the image-text posting format on social media becomes mainstream (Su et al., 2019; Tan and Bansal, 2019), recent studies tend to focus more on multimodal named entity recognition tasks (MNER), to improve the accuracy of text feature extraction with the help of feature elements in images.

### 2.2. Multimodal named entity recognition

Moon et al. (2018) transformed the NER task into a sequence annotation problem with character embeddings and word embeddings as text data inputs and a weighted combination of textual and visual information through an adaptive co-attentive network. Lu et al. (2018) proposed a salient visual attention model to find image regions associated with textual content. The visual contextual information is extracted and fused into the word-level output of the biLSTM model. Zhang et al. (2018) designed an adaptive co-attentive network layer to simultaneously learn the fused feature vectors of vision and language. Moreover, a filtering gate was designed to determine whether the fused features contribute to the accuracy of the annotation task. Arshad et al. (2019) also proposed a gated multimodal fusion representation, where gated fusion is a weighted sum of visual attention features and marker alignment features. Whereas, additive attention scores between word queries and picture features were used to weigh and calculate the visual attention features using VGG-19 visual features (Simonyan and Zisserman, 2014). Chen S. et al. (2020) and Chen X. et al. (2021) extracted visual information into subtitles and proposed a softer method of image-text combination that improves the fusion of different modal features.

### 2.3. Visual-linguistic pre-training

Visual-linguistic pre-training mainly learns the semantic correspondence between different modalities by pre-training on large-scale data to achieve proper operation of the model in resource-poor scenarios. There are already many models implemented based on single-stream (Murahari et al., 2020; Hong et al., 2021) and dual-stream (Gan et al., 2020; Gao et al., 2020) architectures and pre-trained on common datasets such as COCO (Lin et al., 2014). Many VLP models use a single encoder architecture, where the multimodal fusion representation is fed directly to the output layer to generate the final output. In contrast, other VLP models advocate the use of the encoder–decoder architecture commonly used by Transformer, where the multimodal representation is first fed to the decoder and then to the output layer.

Overall, the key issues of the current multimodal named entity recognition task remain in the strategy of combining visual features with linguistic features (Li et al., 2022) and the subsequent entity extraction architecture (He et al., 2016; Li et al., 2020). Therefore, in this study, we explore a multi-channel and multi-level named entity recognition architecture to accomplish MNER for text-image modal tweets.

## 3. Methodology

**Figure 2:**
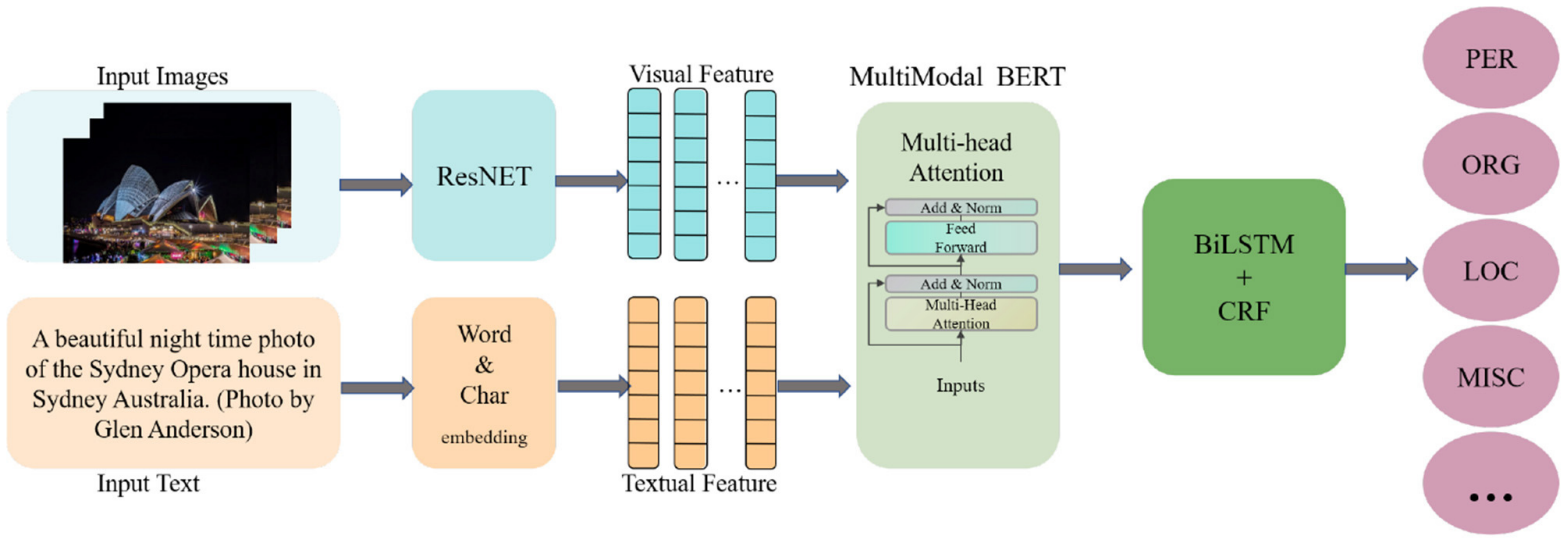
Overall architecture of our MLNet.

The overall structure of the MLNet model roughly consists of several components (shown in Figure 2): image feature extraction, text embedding, multimodal fusion embedding, and label prediction. We first perform image feature extraction, and subsequently input the overall image features and focused relevant visual information into the multimodal BERT along with the text vector to obtain a sequence vector containing rich entity information. Subsequently, contextual information is further learned in the BiLSTM structure. CRF are used to perform the final label prediction.

### 3.1. Image feature extraction

In the case where an image is associated with the corresponding text content, the named entities mentioned in the text are often associated with only the salient features in the image. For the original images in the dataset, the top *s* salient local visual objects in them are first extracted using a pre-trained target detection model. Then, we align the global and local images by scaling so that the size of each image is 224 × 224 pixels, noted as the global image *L*_0_ and local image *L* = {*L*_1_, *L*_2_, ..., *L*_*s*_}.

**Figure 3:**
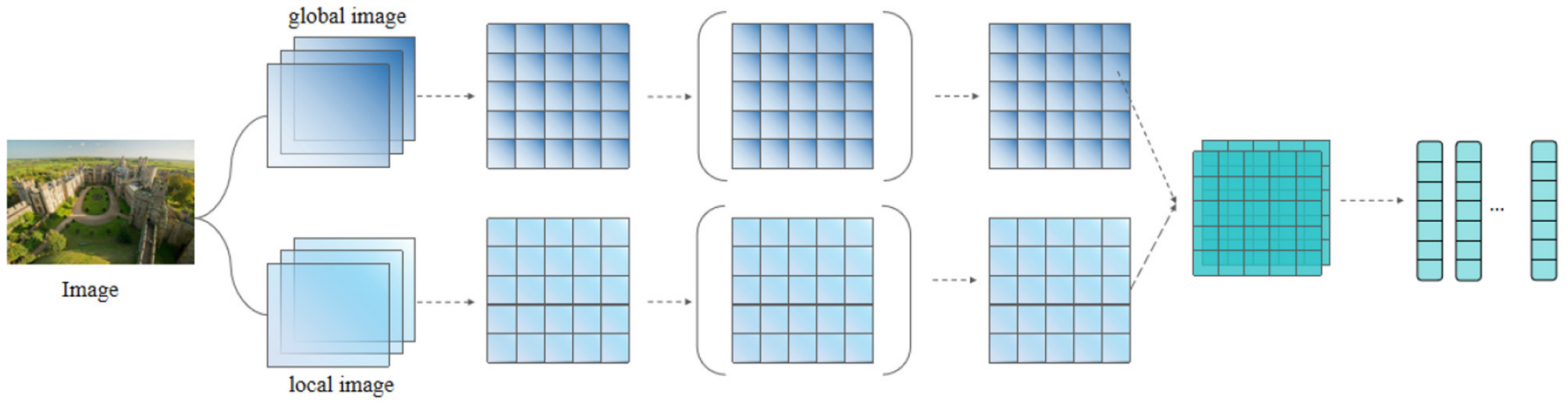
Image feature extraction.

Features were subsequently extracted from the images *L* and *L*_0_ using ResNet as the image feature input for the entire subsequent named entity recognition network architecture. Considering the excellent performance of the ResNet network in object detection tasks, we hypothesize that residual networks can also effectively recognize entities that may appear in images, thereby reducing the impact of image noise on entity recognition accuracy. We use a lightweight ResNet structure with 18 convolutional layers, where each layer uses a convolutional kernel, and residual blocks are used between each convolutional layer to increase the depth of the model. The last pooling layer feature in ResNet is retained with dimension 7 × 7 × *d*, where 7 × 7 is the 49 regions of the image and *d* = 2048 is the dimension of each visual region. At this point, each image can be represented as $\tilde{\nu_{I}}=\left\{\tilde{\nu_{i}}|\tilde{\nu_{i}}\in {\mathbb{R}}^{},i=1,2,...,49\right\}$, and it is subsequently linearly varied and normalized to maintain the same dimensionality as the text vector.


(1) $$\begin{array}{l} \nu_{I}=tanh\left(W_{I}\cdot \tilde{\nu_{I}}+b_{I}\right) \end{array}$$

where the parameters *W*_*I*_ and *b*_*I*_ can be obtained from the training data. The image features obtained at this point can be used as input to the subsequent overall recognition framework to be combined with text features. The visual feature pre-processing process is shown in Figure 3.

### 3.2. Image-text feature fusion

**Figure 4:**
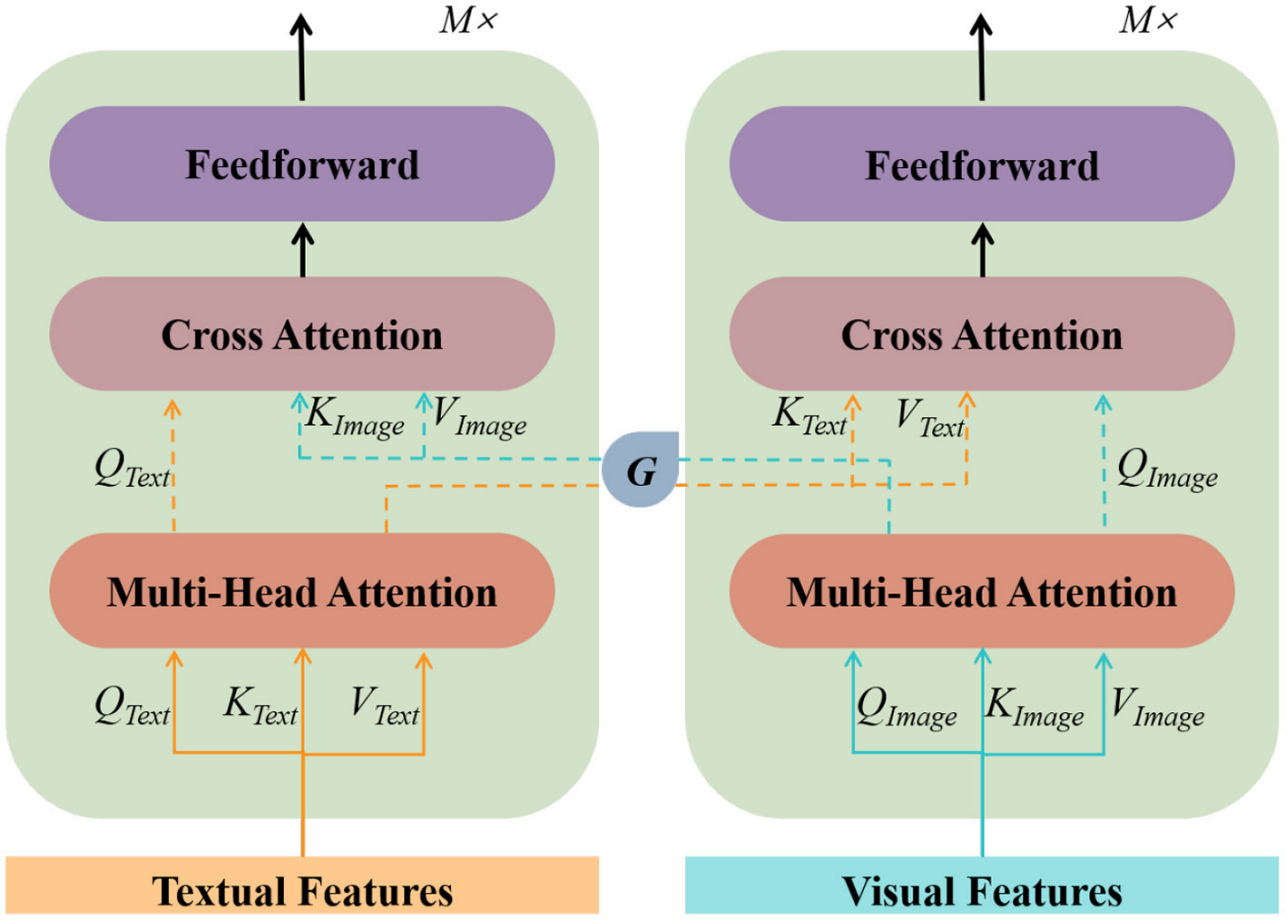
Multi-modal BERT architecture.

Taking into account both the complexity of the model and the actual effectiveness, we chose the WordPiece tokenizer used in BERT for tokenization and word embedding. For character embedding, we adopted a basic recurrent neural network (RNN) model. After completing image and text data pre-processing separately, we choose a BERT structure capable of handling multimodal inputs to realize the joint representation of text and images. The text vectors in the dataset (which have completed word embedding and character embedding) and their corresponding set of image vectors (including global images and local images) are sent to the image converter and text converter independently as inputs to the multimodal BERT. The two converters are trained separately and do not share parameters, and the converters are implemented by a multi-head attention mechanism as follows:


(2) $$\begin{array}{l} MultiHead\left(Q_{F},K_{F},V_{F}\right)=Concat\left(head_{1}^{F},...,head_{n}^{F}\right)W^{O} \end{array}$$

(3) $$\begin{array}{l} Head_{i}^{F}=Attention\left(QW_{i}^{Q_{F}},KW_{i}^{K_{F}},VW_{i}^{V_{F}}\right) \end{array}$$

where *Q* is the query matrix, *K* is the key matrix, *V* is the value matrix, *W* is the weight matrix, and *F* = {*Text, Image*} is used to distinguish the image conversion module from the text conversion module. The multi-head attention mechanism projects *Q*, *K*, and *V* through several different linear transformations, and finally, stitches the different attention results together. To improve the task efficiency, we add a cross-attention layer for cross-modal interaction and introduce a control unit to discard the visual information that is less relevant to the text. The main structure of our multimodal BERT is shown in Figure 4.

For the multimodal BERT structure, we choose three common target tasks for pre-training as follows: (i) masked language modeling (MLM); (ii) masked vision modeling (MVM); and (iii) visual-language matching (VLM). In task, (i) some elements of the text input are masked, but vectors corresponding to image regions are not masked. Task (ii), in contrast to (i), partially masks the image input, but the corresponding text vectors are not masked. In the MVM task, we mark all masked pixel values in the image as a special value of −1 to distinguish them from the original colored image.

The MLM task can be expressed as follows:


(4) LMLM=−E(v,w)~DlogP(wm|wm,v)

where **v** denotes visual, **w** denotes text, **w**_*m*_ denotes masked text, **w**_*m̸*_ denotes remained text, and *D* denotes the training data set.

The MVM task can be expressed as follows:


(5) LMVM=E(v,w)~Df(vm|vm,w)

We choose label classification as the MVM pre-training task, where the mask features are fed into the FC layer to predict the category scores of the objects and then normalized using the softmax function. The detected object categories are used as labels for the masked regions as follows:


(6) f1(vm|vm,w)=∑i=1KCE(c(vmi)−g1(vmi))

where $g_{1}\left({\text{v}}_{m}^{i}\right)$ is the class of detected objects, and *K* denotes the number of visual areas.

We will minimize the KL dispersion (Chen Y.-C. et al., 2020) between the two distributions of the real object class and the detected object class to complete the supervised learning, which is the original output of the detector as follows:


(7) f2(vm|vm,w)=∑i=1KKDL(c^(vmi)−g2(vmi))

where $g_{2}\left({\text{v}}_{m}^{i}\right)$ is the distribution of detected object classes.

By performing the above two tasks, we complete the training of the transformer for each of the image module and text modules. Before the cross-attention layer, we fuse the keys *K* and values *V* of the image and text separately to achieve more fine-grained feature interactions. We use fully connected layers to perform the fusion process. The fused representation of the two patterns is then provided to the FC layer and the sigmoid function to predict a score between 0 and 1, where 0 means visual and verbal mismatch and 1 means visual and verbal match. When the predicted value is less than 0.5, we discard the visual vector because it indicates that the mapping is not relevant enough to the text, and adding visual features at this point will reduce the accuracy of entity recognition.


(8) $$G(K,T)=\{\begin{array}{l} 1\;,\;sigmoid(LN(Text,Image))>0.5\\ 0\;,\;sigmoid(LN(Text,Image))⩽0.5 \end{array}$$

where *LN*(·) denotes the fusion score of image and text, and *G* is the cross-attention layer coefficient.

### 3.3. Label prediction

Since the short text can contain less contextual information, we further use the BiLSTM structure for contextual encoding, in order to preserve the complete semantics as much as possible. The core of the LSTM consists of the following structures: the forgetting gate, the input gate, the output gate, and the memory cell; the common function of the input gate and the forgetting gate is to discard the useless information and pass the useful information to the next moment. The output of the whole structure is obtained by multiplying the output of the memory cell and the output of the output gate. Since the one-way LSTM model cannot handle the contextual information at the same time, the BiLSTM (Bidirectional Long-Short Term Memory) proposed by Graves A et al. The basic idea is to take forward and backward LSTM for each input sequence, respectively, and then, the outputs of the same moment are merged. Thus, for each moment, there corresponds to forward and backward information, which can be expressed as $h_{t}=\left[\vec{h_{t}},\overset{⃖}{h_{t}}\right]$, where $\vec{h_{t}}$ and $\overset{⃖}{h_{t}}$ denote the forward and backward outputs of the bi-directional LSTM, respectively. In the named entity recognition task, BiLSTM is good at handling long-range textual information but cannot handle the dependencies between neighboring labels. Moreover, CRF can obtain an optimal prediction sequence by the relationship of neighboring labels, which can compensate for the shortcomings of BiLSTM.

For the input sequence *X* = {*x*_1_, *x*_2_, ..., *x*_*n*_}, it is assumed that *P*∈ℝ^*n*×*k*^ is the output score matrix of the BiLSTM, where n is the input vector dimension, k is the number of labels, and the score of the *j*−th label of the *i*−th word. For the prediction sequence *Y* = {*y*_1_, *y*_2_, ..., *y*_*n*_}, the formula to calculate its score is as follows:


(9) $$s\left(X,Y\right)=\sum_{i=0}^{n}A_{y_{i},y_{i+1}}+\sum_{i=1}^{n}P_{i,y_{i}}$$

Where *A* denotes the matrix of transferred scores, *A*_*ij*_ representing the scores transferred from label *i* to label *j*. The size of *A* is *k*+2. The probability of generating the predicted sequence *Y* is as follows:


(10) $$\begin{array}{l} p(Y|X)=\frac{e^{s(X,Y)}}{\sum_{\tilde{Y}\in Y_{X}}s(X,\tilde{Y})} \end{array}$$

The likelihood function of the predicted sequence is obtained by taking the logarithm on both sides as follows:


(11) $$\begin{array}{l} ln\left(p\left(Y|X\right)\right)=s\left(X,Y\right)-ln\left(\underset{\tilde{Y}\in Y_{X}}{\sum }s\left(X,\tilde{Y}\right)\right) \end{array}$$

where *X* denotes the true labeled sequence and *Y* denotes all possible labeled sequences. The output sequence of the maximum score is obtained after decoding as follows:


(12) $$\begin{array}{l} Y^{{}^{\circ}}=\underset{\tilde{Y}\in Y_{X}}{\text{argmax}}s\left(X,\tilde{Y}\right) \end{array}$$

## 4. Experiment

### 4.1. Experiment settings

This study uses two publicly available Twitter datasets, Twitter2015 and Twitter2017, constructed by Zhang et al. (2018) and Lu et al. (2018), respectively. These two datasets mainly include multimodal user posts posted on Twitter during 2014–2015 and 2016–2017. Table 1 shows the number of entities and multimodal tweet counts for each type in the training, development, and testing sets for both datasets.

**Table 1:** The basic statistics of our two Twitter datasets.

**Entity type**	**Twitter2015**			**Twitter2017**		
	**Train**	**Dev**	**Test**	**Train**	**Dev**	**Test**
Person	2,217	552	1,816	2,943	626	621
Location	2,091	522	1,697	731	173	178
Organization	928	247	839	1,647	375	395
Miscellaneous	940	225	726	701	150	157
Total	6,176	1,546	5,078	6,049	1,324	1,351
Num of Tweets	4,000	1,000	3,257	3,373	723	723

**Evaluation system:** The common labeling systems for named entity identification are the BIO system, BIOE system, and BIOES system, and the BIO system is chosen in this study. The system has nine labels, namely, “O”, “B-PER”, “I-PER”, “B-ORG”, “I-ORG”, “B-LOC”, “I-LOC”, “B-MISC”, and “I-MISC”.

In this study, recall R, precision P, and F1 values are used to judge the performance of the model, and each evaluation index is calculated as follows:


(13) $$\begin{array}{l} P=\frac{a}{B}\times 100\% \end{array}$$

(14) $$\begin{array}{l} R=\frac{a}{A}\times 100\% \end{array}$$

(15) $$\begin{array}{l} F_{1}=\frac{2PR}{P+R}\times 100\% \end{array}$$

Where *a* is the number of correctly identified entities, *A* is the total number of entities, and *B* is the number of identified entities.

### 4.2. Baseline

To exemplify the effectiveness of our new models, we selected several benchmark models for comparison. We first consider a representative set of text-based models as follows: 1) CNN-BiLSTM-CRF, a widely adopted NER neural network model that is an improvement in BiLSTM-CRF that replaces word embeddings with character-level word embeddings and CNN-based concatenation of character-level word representations for each word; 2) BERT- CRF which is a pioneering study that eliminates the heavy reliance on hand-crafted features and simply employs a bi-directional LSTM model and then uses CRF layers for the final prediction of each word.

In addition, we compare other multimodal approaches for named entity recognition as follows: (1) AdapCAN-Bert-CRF (Zhang et al., 2018) which designs an adaptive co-attention network to induce visual representations of each word; (2) VisualBERT (Li et al., 2019) which differs from the above mentioned SOTA based mainly on co-attention approach, VisualBERT is a single-stream structure, which is a strong baseline for comparison; (3) OCSGA (Wu et al., 2020), a model that combines dense co-attentive networks (self-attentive and guided attention), to model the association between visual objects and textual entities and the intrinsic connections between objects or entities; (4) UMT (Yu et al., 2020) by adding a multi modality converter to achieve a unified architecture by adding an auxiliary entity span detection module; (5) UMGF (Zhang et al., 2021), using a multimodal graph fusion approach, captures various semantic relationships between multimodal semantic units (words and visual objects); (6) HVPNet (Chen et al., 2022), using dynamic threshold aggregation strategy to achieve hierarchical multiscale visual features as fused visual prefixes.

In the multimodal BERT structure, we choose 12 layers, each hidden layer has a size of 768 and 12 self-attentive heads. In the training process of the BiLSTM-CRF structure, the Adam optimizer is used, and the learning rate is chosen as 0.001. In addition, the LSTM dimension is set to 200, the batch size is 64, and the maximum vector length of text input is 128, to prevent an overfitting problem. Dropout is used in the input and output of BiLSTM, and the value is 0.5 (Bouthillier et al., 2015).

### 4.3. Main results

In Table 2, we report the precision (P), recall (R), and F1 score (F1) achieved by each method on the two Twitter datasets.

**Table 2:** Performance comparison on our two TWITTER datasets.

**Methods**	**Twitter2015**			**Twitter2017**		
	**Precision**	**Recall**	**F1**	**Precision**	**Recall**	**F1**
CNN-BiLSTM-CRF	66.24	68.09	67.15	80.00	78.76	79.37
BERT-CRF	69.22	74.59	71.81	83.32	83.57	83.44
AdapCAN-BERT-CRF	69.87	74.59	72.15	85.13	83.20	84.10
VisualBERT	68.84	71.39	70.09	84.06	85.39	84.72
OCSGA	74.71	71.21	72.92	-	-	-
UMT	71.67	75.23	73.41	85.28	85.34	85.31
UMGF	74.49	75.21	74.85	86.54	84.50	85.51
HVPNet	73.87	76.82	75.32	85.84	**87.93**	86.87
MLNet (ours)	**75.73**	**76.85**	**76.28**	**87.36**	86.97	**87.17**

The best results in the table are highlighted in bold.

**Figure 5:**
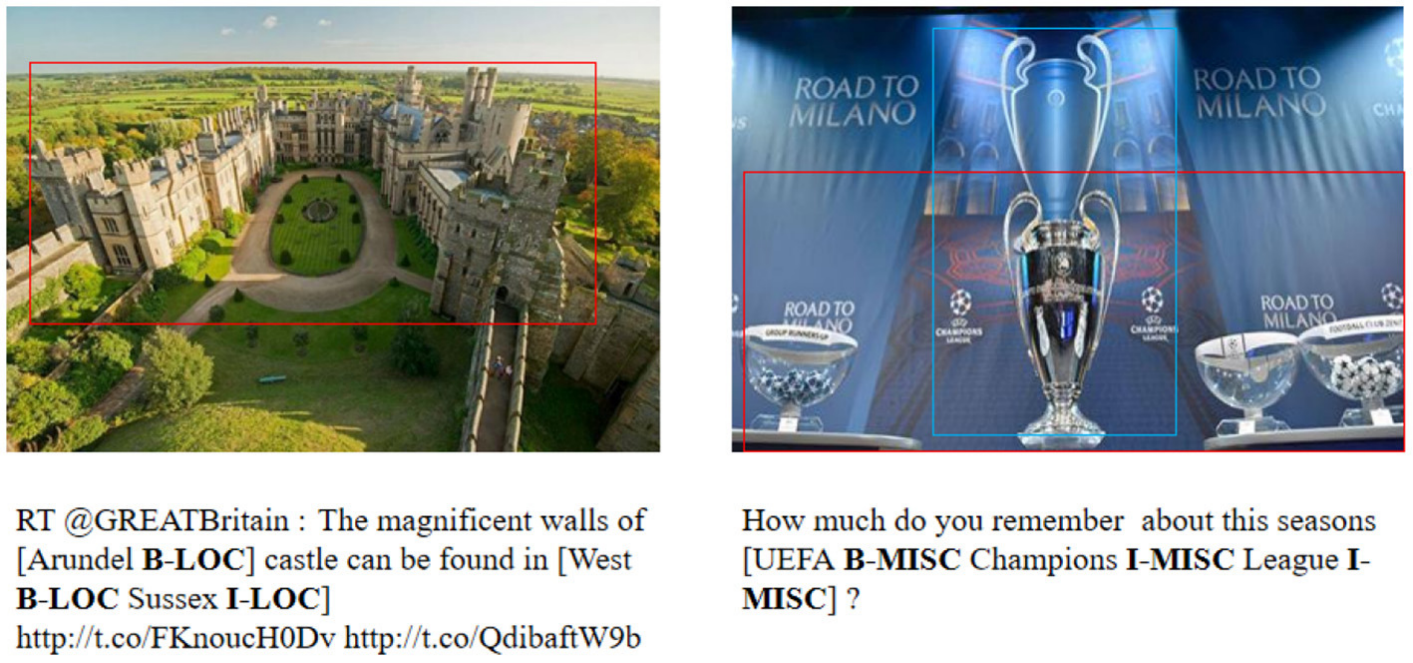
Two examples of correct visual attention. Our model successfully highlights related image regions required to predict correct tag.

First, compared with the two text-based methods CNN-BiLSTM-CRF and BERT-CRF, it is clearly observed that our model outperforms the other methods on both datasets. It is clear that the inclusion of visual features does guide the NER model well in discovering named entities when solving the named entity recognition task for tweet text. Although the accompanying images of some tweets may not be directly related to the text content, to a certain extent, extracting the corresponding image information (shown as Figure 5) can address the ambiguity issues and irregular representations present in the text.

Second, our model outperforms OCSGA, UMT, and UMGF compared with existing multimodal methods, thus it can be shown that the visual information preprocessed by focused feature extraction is more helpful for text entity extraction task effect enhancement than the complete image. By drawing on the target detection algorithm in the image preprocessing process, we are able to focus more actively on the entity information originally present in the image in the subsequent label classification task, thus avoiding a large amount of irrelevant information interference. Moreover, compared with the pre-trained model VisualBERT in which images and complete text are directly input to BERT for encoding and decoding, our model also introduces character-level embedding to address spelling errors and noise of informal expressions and uses a multi-head attention mechanism to learn different levels and modalities of input information.

Finally, comparing all named entity recognition methods, we can see that our multimodal entity recognition architecture achieves the best results, 0.29 and 1.86% higher than the second best method, respectively, and outperforms the twitter2015 dataset in twitter2017. This indicates that our model is accurate and effective in small-sample scenarios. In this regard, we analyze that our model is more enough to better learn the association between visual features and text features in small sample scenarios, thus improving the accuracy of tag classification.

### 4.4. Further analysis

#### 4.4.1. Ablation study

To understand the role of network structure in the model, we performed more experiments for some variants of our model. For each dataset, we compared the full model MLNet and two variants of the model MLNet w/o Early Fusion and MLNet w/o Mtla. The obtained knots are shown in Table 3.

**Table 3:** Ablation study of MLNet.

**Methods**	**Twitter2015**			**Twitter2017**		
	**Precision**	**Recall**	**F1**	**Precision**	**Recall**	**F1**
MLNet	**75.73**	**76.85**	**76.28**	**87.36**	**86.97**	**87.17**
MLNet w/o Fus.	74.44	73.07	73.75	85.10	84.51	84.80
MLNet w/o Mtla.	74.73	73.72	74.22	85.97	85.62	85.79

The best results in the table are highlighted in bold.

**MLNet:** Complete multi-level multimodal named entity recognition model, including image-text pre-processing, multimodal feature fusion already and feature annotation structure.

**MLNet w/o Fus.:** Instead of combining the image feature vectors extracted by ResNet with the text in the encoder Transformer in BERT, they are combined with the text vectors in the last layer of the Transformer structure before output. This allows us to test whether the interaction between linguistic and visual in the whole Transformer stack is important for performance. This variant structure is able to represent the impact of image features fused with text features for entity recognition tasks.

**MLNet w/o Mtla.:** When encoding text, only word-level vectors are kept, and no character-level feature vectors are introduced. This variant can help us to recognize the help of multi-level structure for improving accuracy.

As we can see in Table 3, the introduction of visual features and character-level embedding vectors can effectively improve the quality of the task. In this regard, we analyze that image information can enhance the semantic understanding of MLNet, and this multimodal BERT structure we use can filter the visual information, retain the regions with higher similarity to the text, and achieve image-text and feature fusion to avoid directly superimposing the less relevant image information on top of the text, which causes unnecessary errors. The introduction of character-level embedding is another important reason for achieving the MLNet effect. Due to the short text length of tweets, it is difficult to obtain the semantics according to the context, and there are certain misspellings, URL addresses, and some emojis, which bring interference with label classification. Preserving character-level embedding can obtain more semantic information to a certain extent and improve the effectiveness of short text recognition tasks.

#### 4.4.2. Cross-task scenario

We further tested the performance of UMGF and MLNet in cross-domain scenarios and compared them. We used the model obtained by training on the Twitter2015 dataset to test Twitter2017 and notated as Twitter2015 → Twitter2017. Similarly, Twitter2017 → Twitter2015 indicates the use of a model trained on Twitter2017 to test Twitter2015. As shown in Table 4, our MLNet achieves better results in terms of F1 Score in this cross-task scenario experiment. This proves that our MLNet has made some progress in model liability.

**Table 4:** Performance comparison of MLNet and UMGF in cross-task scenario.

**Methods**	**Twitter2015**→**Twitter2017**			**Twitter2017**→**Twitter2015**		
	**Precision**	**Recall**	**F1**	**Precision**	**Recall**	**F1**
UMGF	67.00	**62.81**	66.21	69.88	56.92	62.74
MLNet	**70.60**	62.46	**66.28**	**71.01**	**58.40**	**64.09**

The best results in the table are highlighted in bold.

Although MLNet is slightly less effective on the Twitter2015 dataset than the Twitter2017 dataset, our model trained on the Twitter2015 dataset is still more effective than the model trained on the Twitter2017 dataset in the migration experiment. This also shows that although our models have better results on small datasets, training MLNet on larger amounts of data is still effective and can improve the understanding of the models. This cross-migration scenario is interesting to facilitate the entity recognition task and better improve the language model.

## 5. Conclusion

In this study, we propose a new multilevel multichannel fusion network for the named entity recognition task in social media postings. Specifically, we propose a focused visual feature preprocessing method in multimodal tasks to extract visual features related to text semantics as auxiliary inputs, which is an effective visual enhancement of the NER attention module. We also propose the inclusion of character embedding, which expands the feature information that can be extracted from short texts and implements an entity extraction architecture with multiple levels of input. Achieving a better extractor through effective visual enhancement, extensive experiments, and results on three criteria demonstrate the effectiveness and robustness of our proposed approach. At the same time, our method faces the limitation of slower operations. In future, we plan to (1) further investigate the simplified structure of multimodal multilevel entity extraction models to make them more flexible and scalable and (2) try more diverse image-text feature fusion algorithms to help the models better understand the association between visual features and text features.

## Data availability statement

The original contributions presented in the study are included in the article/supplementary material, further inquiries can be directed to the corresponding author.

## Author contributions

HZ and ZH contributed to conception and design of the study. XT organized the database. XL funded acquisition. HZ, ZH, and XT performed the statistical analysis. HZ wrote the first draft of the manuscript. ZH and FB wrote the sections of the manuscript. All authors contributed to manuscript revision, read, and approved the submitted version.
